# Prophylactic intravenous norepinephrine for the prevention of hypotension during spinal anesthesia for elective cesarean section: a systematic review and dose–response meta-analysis of randomized controlled trials

**DOI:** 10.3389/fphar.2023.1247214

**Published:** 2023-09-19

**Authors:** Yuan Li, Bingxing Shuai, Han Huang

**Affiliations:** ^1^ Department of Anesthesiology and Key Laboratory of Birth Defects and Related Diseases of Women and Children, Ministry of Education, West China Second University Hospital of Sichuan University, Sichuan University, Chengdu, China; ^2^ Department of Medical Affairs, West China Hospital, Sichuan University, Chengdu, China

**Keywords:** cesarean section, spinal anesthesia, hypotension, norepinephrine, dose–response meta-analysis

## Abstract

**Introduction:** In this study, we aimed to evaluate the potential dose–response relationship between prophylactic norepinephrine (NE) infusion rates and the risks of hypotension during cesarean section following spinal anesthesia.

**Methods:** Randomized controlled trials with two or more NE doses for post-spinal hypotension prophylaxis during cesarean section were systematically searched in the MEDLINE, Embase, Web of Science, Cochrane Central Register of Controlled Trials, and US Clinical Trials Registry databases until 31 July 2022. The primary outcome was the relative risk of maternal hypotension with different NE regimens (infusion rates or bolus doses). Secondary outcomes included the relative risks of maternal and fetal adverse events with different NE regimens.

**Results:** Ten studies with 1,144 parturients were included for final analysis using restricted cubic splines and random-effects dose–response meta-analysis models. A significant dose–response relationship existed between NE infusion rates and the relative risks of maternal hypotension. Every 0.01 μg/kg/min increment in the NE infusion rate was associated with a 14% decrease in the incidence of post-spinal hypotension. ED_50_ and ED_95_ of NE infusion rates for post-spinal hypotension prophylaxis were estimated to be 0.046 (95% CI from 0.032 to 0.085) and 0.2 (95% CI from 0.14 to 0.37) μg/kg/min, respectively. However, a higher NE infusion rate was associated with a higher incidence of maternal hypertension.

**Conclusion:** An increased NE infusion rate was associated with a decreased incidence of post-spinal hypotension but an increased incidence of hypertension. Therefore, 0.07 μg/kg/min was recommended as the initial NE infusion rate for clinical practice, as it was associated with the lowest risk of physician intervention for unstable hemodynamics after spinal anesthesia for cesarean delivery.

**Systematic Review Registration:** (https://www.crd.york.ac.uk/PROSPERO/display_record.php?RecordID=349934), identifier (CRD42022349934).

## 1 Introduction

Spinal anesthesia or combined spinal–epidural anesthesia (CSEA) is the preferred anesthetic technique for cesarean section (Strouch et al., 2015). Post-spinal hypotension remains the most common adverse event associated with this technique, affecting up to 80% of mothers if no preventative strategy is used (Fitzgerald et al., 2020). Post-spinal hypotension not only causes maternal nausea and vomiting but also leads to severe complications, such as maternal circulatory collapse or fetal acidosis (Ilies et al., 2012). Due to the poor efficacy of non-pharmacological interventions, prophylactic vasopressor infusion is now recommended as the first-line strategy for preventing post-spinal hypotension during cesarean section (Kinsella et al., 2018).

Currently, phenylephrine, a pure α-adrenergic agonist, is recommended as the vasopressor of choice (Author Anonymous, 2016). However, concerns have been raised as phenylephrine causes reflex bradycardia, which may further lead to decreased maternal cardiac output. Over the past few years, norepinephrine (NE) was introduced as a potent α-adrenergic agonist plus weak β-adrenergic agonist for post-spinal hypotension prevention during cesarean section as it caused less decrease in the maternal heart rate than phenylephrine (Ngan Kee et al., 2015; Vallejo et al., 2017). Therefore, it is considered an alternative to phenylephrine to prevent post-spinal hypotension prophylaxis during cesarean section (Ngan Kee, 2017). As different regimens were used in previous optimal infusion rate-finding trials, the estimated ED_50_ and ED_95_ of NE dosages for post-spinal hypotension prophylaxis were different between these studies. Therefore, we aimed to perform this dose–response meta-analysis to determine the best NE infusion regimen for preventing post-spinal hypotension during cesarean section (Desquilbet and Mariotti, 2010; Orsini et al., 2012).

## 2 Methods

### 2.1 Registration and protocol

The study protocol of this systematic review and meta-analysis was registered in the PROSPERO database (ID: CRD42022349934). This review adhered to the Preferred Reporting Items for Systematic Reviews and Meta-Analyses (PRISMA) guidelines (Page et al., 2021).

### 2.2 Search strategy

Two investigators (Y.L and Bx. S.) independently searched EBM Reviews—Cochrane Central Register of Controlled Trials < till June 2022>, Embase <1974 to 2022 July 22>, Ovid MEDLINE(R) and Epub Ahead of Print, In-Process, In-Data-Review & Other Non-Indexed Citations, Daily and Versions <1946 to July 22, 2022>, Web of Science (from 1900 through July 2022). We also reviewed the US Clinical Trials Registry (http://www.clinicaltrials.gov) from inception to the end of July 2022 for in-progress or completed clinical trials that met our inclusion criteria. The Medical Subject Headings and text terms used in the searches reflected the NE and cesarean section concepts. The bibliographies of retrieved articles were reviewed to identify additional references. Only articles in English were included for further assessment. The detailed search strategy for MEDLINE (Ovid) is available in the Supplementary Material.

### 2.3 Study selection

Two authors (YL. and Bx. S.) independently reviewed the retrieved literature works identified by our search strategy described previously. Discrepancies were resolved by discussing or referring to the third author (H.H.) when necessary. Studies that met the following criteria according to the PICOS principle were included (Brown et al., 2006):

(1) *Population*, pregnant women receiving spinal anesthesia for elective cesarean section.
(2) *Interventions*, two or more different NE bolus dosages or infusion rates were used.
(3) *Comparison*, saline control or the minimal NE dose.
(4) *Outcomes*, the primary outcome was the relative risk of maternal hypotension with different NE regimens (infusion rates or bolus doses). Secondary outcomes included the relative risks of maternal hypertension, maternal nausea or vomiting, fetal acidosis, umbilical arterial blood gas status, and Apgar scores with different regimens.
(5) *Study design*, randomized controlled trial (RCT).

Exclusion criteria included a dose-finding study using the up-and-down procedure, hypotension rescued with intravenous NE infusion, and any completed trials or conference articles that did not report required outcomes.

### 2.4 Data extraction

Data were extracted independently by the same two reviewers (Y.L. and Bx. S.) using a pre-designed standardized data collecting form. The following variables were collected: author, year of publication, the country where the study was conducted, study design, sample size, details of anesthesia, definitions of maternal hypotension and hypertension, NE bolus dosages, NE infusion rates, maternal outcomes (maternal hypotension, hypertension, and nausea or vomiting), and neonatal outcomes (umbilical cord arterial blood gases, fetal acidosis, and Apgar scores). The study investigators would be contacted for missing data or clarification.

### 2.5 Risk of bias and quality of evidence assessment

According to the Cochrane Risk of Bias Tool (RoB 1) (Higgins et al., 2011), two authors (Y.L. and Bx. S.) independently assessed the included studies’ risks of bias with the following six domains: random sequence generation (selection bias), allocation concealment (selection bias), blinding of participants and personnel (performance bias), blinding of outcome assessment (detection bias), incomplete outcome data (attrition bias), and selective reporting (reporting bias). For each domain, the risk of bias was categorized as low, unclear, or high. We used the Grading of Recommendation, Assessment, Development, and Evaluation (GRADE) guidelines to assess the quality of evidence as high, moderate, low, or very low for five reasons (increased risk of bias, inconsistent results, indirect evidence, imprecision, and publication bias) (Guyatt et al., 2011).

### 2.6 Statistical analyses

The significance level in this study was set at 0.05. All statistical analyses were performed using Stata version 17 (StataCorp, TX, RRID: SCR_012763). If meta-analysis was not feasible, data were simply represented quantitatively. Continuous variables were reported as mean difference (MD) with a 95% confidence interval (CI), while dichotomous data were presented as a risk ratio (RR) with a 95% CI. The primary outcome was the relative risk of maternal hypotension with different NE regimens (infusion rates or bolus doses). The relative risk of maternal hypotension following spinal anesthesia was first calculated between the highest and lowest NE doses/infusion rates in the included RCTs, using the random-effects meta-analysis model proposed by DerSimonian and Laird (1986) (high versus low meta-analysis). Then, the potential non-linear dose–response relationship was explored using restricted cubic splines with three knots in the dose–response regression model by only including RCTs with three or more dose levels (Desquilbet and Mariotti, 2010; Orsini et al., 2012). The results from all these RCTs were then pooled for multivariate random-effects meta-analysis (L et al., 2015). If non-linear trends evaluated by the Wald test were insignificant, the study-specific linear trends between NE dosages/infusion rates and risk of maternal hypotension were estimated using the method described by Greenland and Longnecker (1992). Q or I^2^ was used for heterogeneity evaluation and was considered significant if *p* < 0.05 or I^2^ > 50% (Higgins et al., 2003). Subgroup analyses were performed to assess whether the mode of prophylactic NE administration (bolus injection or continuous infusion) affected the dose–response relationship for hypotension (Sun et al., 2014). A sensitivity analysis using the leave-one-out approach was conducted when significant heterogeneity existed. Publication bias was assessed using an imputed contour-enhanced funnel plot and Egger’s test (Egger et al., 1997). To evaluate the net benefit of prophylactic NE administration for each individual, the benefit and harm of NE administration were estimated by calculating the absolute risk reduction (ARR) and the absolute risk increase (ARI) when a dose–response relationship was found (Lee et al., 2004). To determine the threshold of the NE infusion rate with an equal risk reduction and increase, we graphed the NE infusion rate (x-axis) and the absolute risk difference (y-axis) (Glasziou and Irwig, 1995). Physician intervention for unstable hemodynamics was defined as rescuing vasopressor bolus for hypotension and temporary cessation of NE infusion for hypertension. The dose–response analysis was also performed between physician intervention and NE infusion rate. We performed trial sequential analysis (TSA) including trials with a comparison between NE and saline control. The required information size (RIS) was then estimated, with a type I error of 0.05 and type II error of 0.20. This was performed by employing the random-effects model and assessing heterogeneity through the diversity (*D* ^2^) within the encompassed trials (Wetterslev et al., 2009). TSA was performed using TSA software (Version 0.9.5.10 Beta, Copenhagen Trial Unit, Denmark).

## 3 Results

### 3.1 Study selection

**FIGURE 1:**
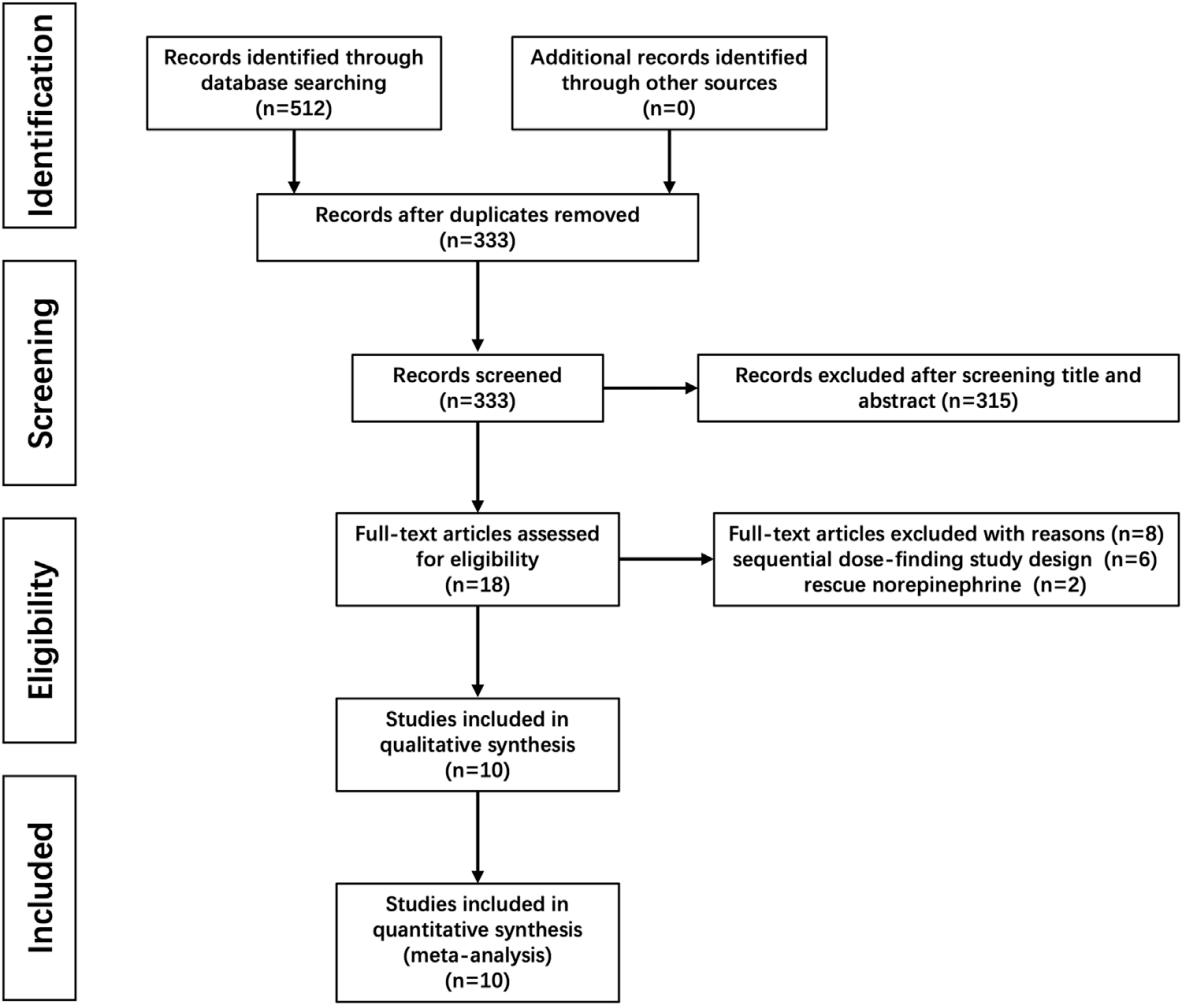
Flow diagram of included studies.

**FIGURE 2:**
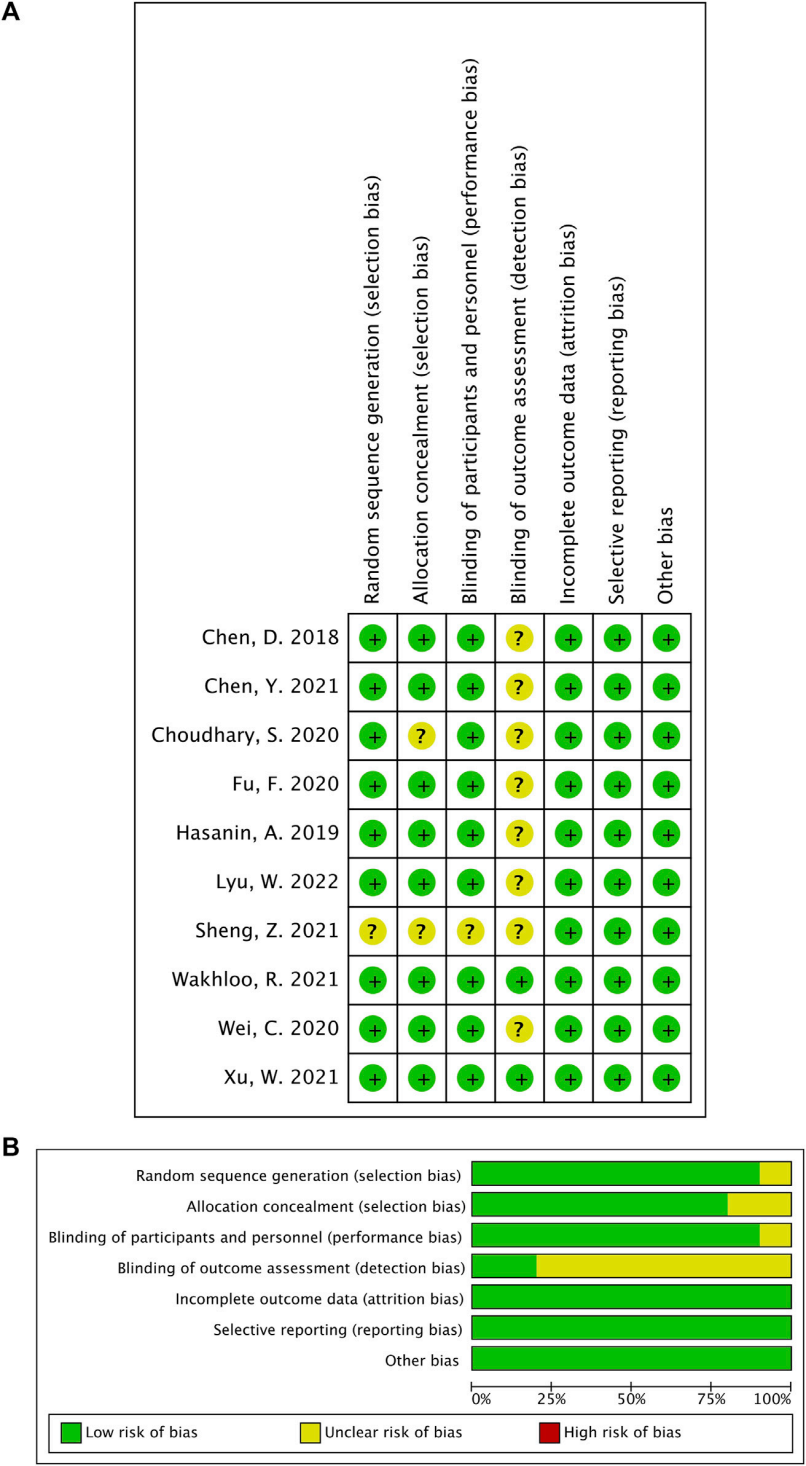
**(A)** Risk of bias graph. **(B)** Risk of bias summary.

A total of 512 citations were identified with our search strategy, and 179 were removed as duplicates. Of the remaining 333 studies, 315 were removed based on the title and abstract screening; six were further excluded because the up-and-down method was used for dose finding; and another two were also excluded as NE was used for rescuing instead of preventing hypotension. Therefore, 10 RCTs were finally included in this systematic review (Chen et al., 2018; Hasanin et al., 2019; Choudhary et al., 2020; Fu et al., 2020; Wei et al., 2020; Chen et al., 2021; Sheng et al., 2021; Wakhloo et al., 2021; Xu et al., 2021; Lyu et al., 2022). The flow diagram for study selection is shown in Figure 1, and the risk of bias for each included study is shown in Figure 2.

### 3.2 Study characteristics

The main characteristics of included RCTs are listed in Table 1. A total of 1,144 parturients were involved in these 10 RCTs. Only singleton pregnancy data were extracted, as twin pregnancies were also recruited in the trial from Sheng et al. (2021). The definition of maternal hypotension varied between studies. In nine of the ten included studies, hypotension was defined as systolic blood pressure (SBP) less than 80% of the baseline value or less than 90 mmHg. However, in one study, it was defined as SBP less than 90% of the baseline level (Choudhary et al., 2020). There were three modes for prophylactic NE administration among the included 10 RCTs: single bolus injection (Choudhary et al., 2020; Wakhloo et al., 2021), continuous infusion (Chen et al., 2018; Fu et al., 2020; Wei et al., 2020; Sheng et al., 2021; Xu et al., 2021), and bolus injection followed by continuous infusion (Hasanin et al., 2019; Chen et al., 2021; Lyu et al., 2022). RCTs were further divided into four subgroups according to different bolus dose and infusion rate combinations: trials with variable infusion rates (Chen et al., 2018; Fu et al., 2020; Wei et al., 2020; Sheng et al., 2021; Xu et al., 2021), trials with fixed bolus dose plus variable infusion rates (Hasanin et al., 2019; Chen et al., 2021), trials with variable bolus doses (Choudhary et al., 2020; Wakhloo et al., 2021), and trials with variable bolus doses plus fixed infusion rate (Lyu et al., 2022).

**TABLE 1:** Characteristics of included studies.

Study	Design	Anesthesia	Norepinephrine (NE) regimen
Lyu et al. (2022)	RCT (n = 115)	Hydration: Ringer’s lactate 20 ml/min co-loaded maximum to 1.5 L	NE was administered during the initiation of intrathecal injection, followed by 0.05 μg/kg/min, and stopped after delivery
	Hypotension: SBP<80% of the baseline value	Position: Supine position	Dose: 0.05 or 0.10 μg/kg
	Severe hypotension: SBP<60% of the baseline value	Anesthetics: Ropivacaine 15 mg	Rescue: 40 or 100 μg phenylephrine
	Bradycardia: Heart rate (HR) < 60 beats per minute		
Xu et al. (2021)	RCT (n = 100)	Hydration: Lactated Ringer’s solution 10 ml/kg co-loaded over 30 min	NE was administered immediately after intrathecal injection at a rate of 50 ml/h and stopped after delivery
	Hypotension: SBP≦80% of baseline or SBP <90 mmHg	Position: 15° left uterine displacement	Dose: 0, 0.025, 0.05, 0.075, and 0.1 μg/kg/min
	Bradycardia: HR < 50 bpm	Anesthetics: Bupivacaine 10 mg and sufentanil 5 μg	Rescue: 50 µg phenylephrine or 4 µg NE, or 6 mg ephedrine under different situations
Wakhloo et al. (2021)	RCT (n = 60)	Hydration: Lactated Ringer’s co-loaded maximum of 1 L	NE given as the sensory block reached the T6 level
	Hypotension: SBP<80% of the baseline	Position: Left lateral tilt of 15–20°	Dose: 10 or 15 μg
	Bradycardia: heart rate<60 beats/min	Anesthetics: Bupivacaine 11 mg	Rescue: 3 mg mephentermine
Sheng et al. (2021)	RCT (n = 100)	Hydration: Preloaded 10 ml/kg of lactated Ringer’s solution and co-hydration with 500 ml hydroxyethyl starch solution over 15–20 min	NE was infused during the initiation of intrathecal injection
	Hypotension: SBP<80% of the baseline	Position: Not specified	Dose: 0.02, 0.03, 0.04, 0.05, and 0.06 μg/kg/min/
		Anesthetics: Ropivacaine 16 mg	Rescue: Not specified
Chen et al. (2021)	RCT (n = 99)	Hydration: Preloaded compound sodium chloride (5 ml/kg) (0.85% NaCl, 0.03% KCl, and 0.033% CaCl_2_) and co-loaded 8 ml/kg/h	6 μg loading bolus of NE or NS following spinal anesthesia and a maintenance dose of NE or NS
	Hypotension: SBP<80% of the baseline value	Position: Not specified	Dose: 0, 0.025, 0.05, 0.075, and 0.1 μg/kg/min
	Severe hypotension: SBP<60% of the baseline	Anesthetics: Bupivacaine 12.5 mg	Rescue: 6 μg NE
	Bradycardia: HR < 55 bpm		
Wei et al. (2020)	RCT (n = 99)	Hydration: Co-loaded lactated Ringer’s solution (5 ml/kg) was administered over 20–30 min	NE was infused concurrent with the intrathecal injection
	Hypotension: SBP≦80% of baseline or SBP <90 mmHg	Position: Left uterine displacement	Dose: 0, 0.04, 0.05, 0.06, and 0.07, μg/kg/min
	Bradycardia: HR < 50 bpm	Anesthetics: Bupivacaine 10 mg and sufentanil 5 μg	Rescue: 6 mg of ephedrine
Fu et al. (2020)	RCT (n = 80)	Hydration: Co-loaded with lactated Ringer’s solution to a maximum of 1.5 L	NE was administered immediately after intrathecal injection
	Hypotension: SBP≦80% of baseline or SBP <90 mmHg	Position: Left uterine displacement	Dose: 0.025, 0.05, 0.075, 0.10 μg/kg/min
	Bradycardia: HR < 50 beats/min	Anesthetics: Ropivacaine 15 mg	Rescue: SBP >90 mmHg, rapid infusion of lactated Ringer’s solution, SBP <90 mmHg, 50 μg phenylephrine
Choudhary et al. (2020)	RCT (n = 90)	Hydration: Preloaded with Ringer’s lactate solution at 10 ml/kg/h over 30 min	NE was administered along with the subarachnoid block
	Hypotension: SBP<90% of baseline	Position: Left uterine displacement	Dose: 0, 4, and 6 μg
	Bradycardia: Heart rate<50 beats/min	Anesthetics: Bupivacaine 10 mg	Rescue: 6 μg NE.
Hasanin et al. (2019)	RCT (n = 284)	Hydration: Crystalloid co-loaded to a maximum of 1.5 L	5 μg NE bolus followed a maintenance dose of NE
	Hypotension: SBP<80% of the baseline value	Position: Left uterine displacement	Dose: 0.025, 0.050, and 0.075 μg/kg/min
	Severe hypotension: SBP<60% of the baseline	Anesthetics: Bupivacaine 10 mg and fentanyl 20 μg	Rescue: SBP<80% baseline, 9 mg ephedrine, SBP<60% baseline, 15 mg ephedrine
	Bradycardia: HR < 55 bpm		
Chen et al. (2018)	RCT (n = 117)	Hydration: Preloaded with lactated Ringer’s solution of 10 ml/kg within 20 min, maintained with 20 ml/min	NE was infused immediately after intrathecal injection
	Hypotension: SBP≦80% of baseline or SBP <90 mmHg	Position: Tilted supine position	Dose: 0, 5, 10, and 15 μg/kg/h
	Bradycardia: heart rate <50 beats/min	Anesthetics: Ropivacaine 11–12.5 mg and morphine 0.1 mg	Rescue: 10 μg NE.

### 3.3 Outcomes

#### 3.3.1 Primary outcome

**FIGURE 3:**
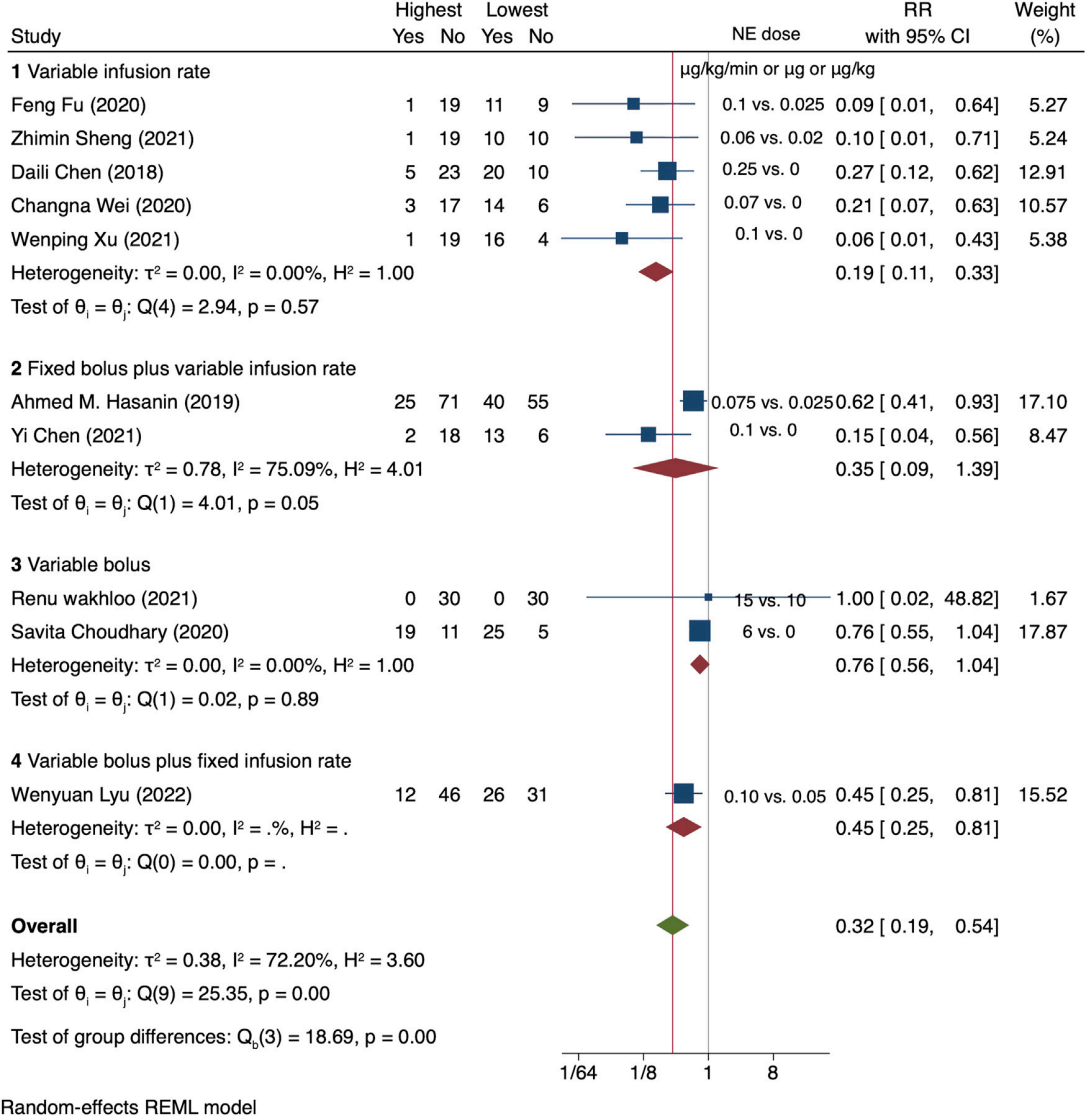
Summary of relative risk of maternal hypotension in different regimens, the highest vs. the lowest dose category.

For high versus low meta-analysis, all the 10 RCTs were included. The relative risk of maternal hypotension between the highest and the lowest NE doses was 0.32 (95% CI from 0.19 to 0.54), with significant heterogeneity (I^2^ = 72.2%, *p* < 0.001) (Figure 3).

**FIGURE 4:**
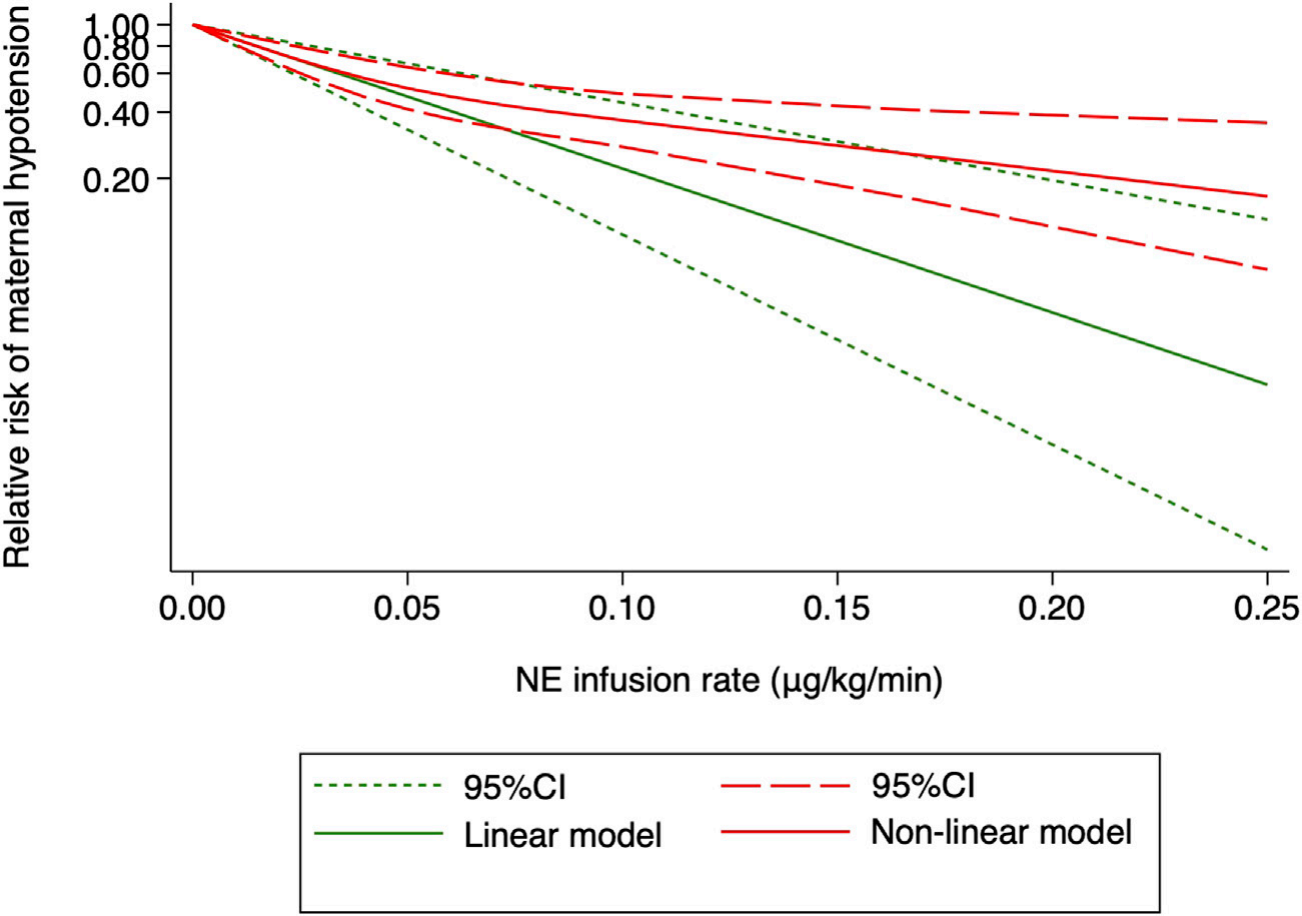
Dose–response relationship between the NE infusion rate and relative risk of maternal hypotension, showing point estimates and 95% CI for linear and non-linear meta-analysis models.

A dose–response relationship was observed between NE infusion rates and relative risks of maternal hypotension in both linear and non-linear models with random effects (*X* ^2^ = 18.27 and 50.14, respectively, *p* < 0.001 for both models). The pooled relative risk of maternal hypotension was 0.86 (95% CI from 0.80 to 0.92, *p* < 0.001) with every 0.01 μg/kg/min increase in the NE infusion rate (Figure 4). The estimated ED_50_ and ED_95_ of NE infusion rates for post-spinal hypotension prophylaxis were 0.046 (95% CI from 0.032 to 0.085) and 0.20 (95% CI from 0.14 to 0.37) μg/kg/min, respectively.

**FIGURE 5:**
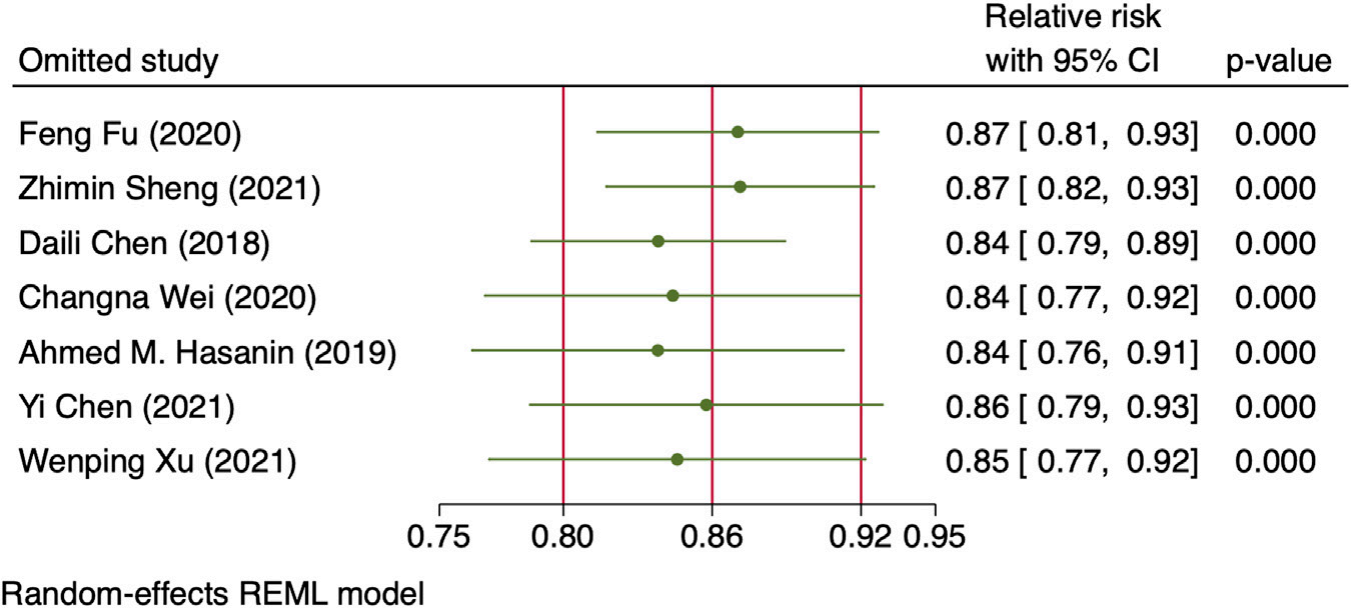
Sensitivity analysis of individual studies influenced the pooled relative risk of maternal hypotension per 0.01 μg/kg/min increase in the NE infusion rate.

**FIGURE 6:**
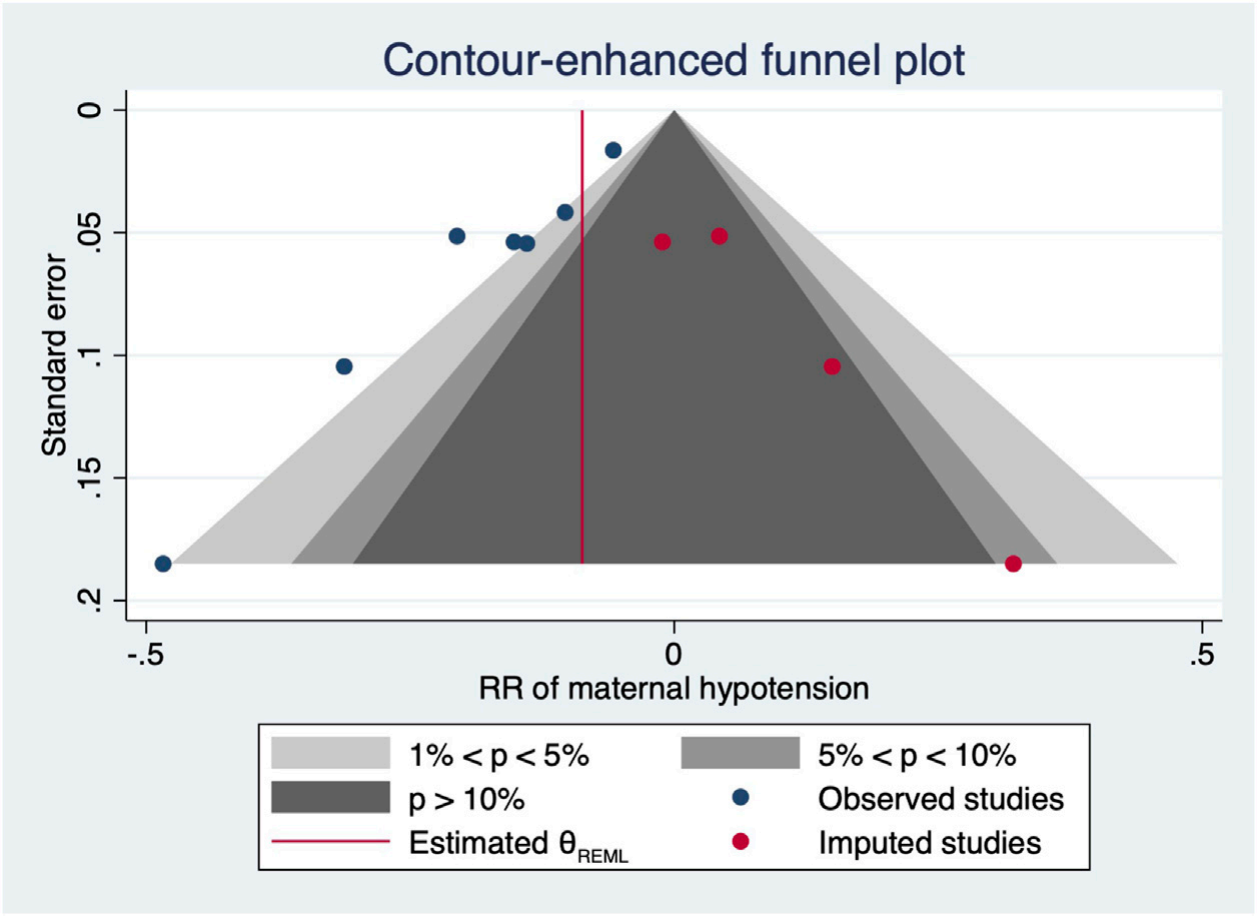
After imputation, the contour-enhanced funnel plot of maternal hypotension.

**FIGURE 7:**
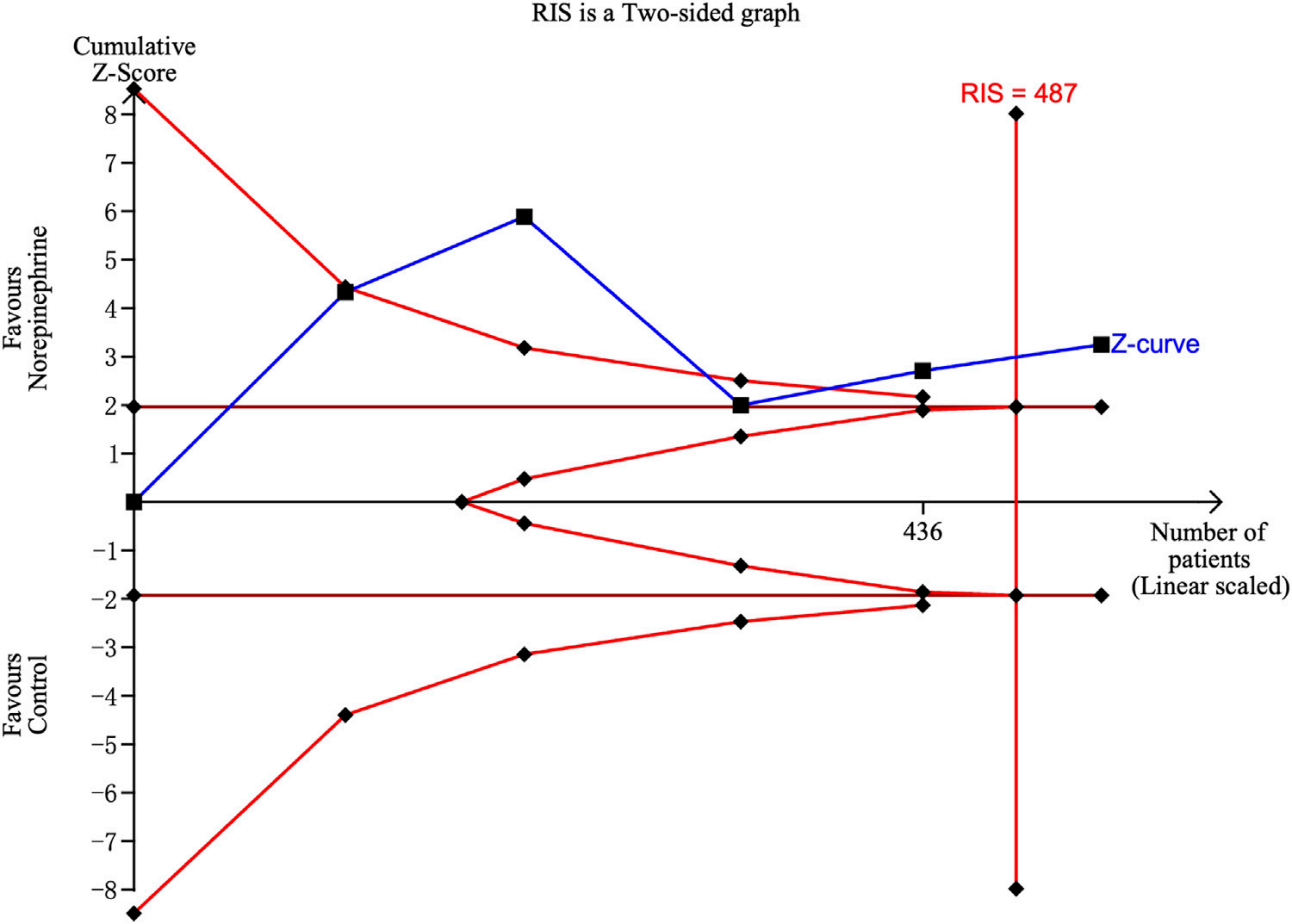
Trial sequential analysis on the incidence of maternal hypotension in studies comparing NE with the saline control.

However, there were significant heterogeneities across included RCTs (*X* ^2^ = 20.27 and 34.91 with *p*-values = 0.0025 and 0.0396 for linear and non-linear models, respectively). Subgroup analysis was then performed to reduce heterogeneity caused by different modes of NE administration by including trials with variable infusion rates only (Chen et al., 2018; Fu et al., 2020; Wei et al., 2020; Sheng et al., 2021; Xu et al., 2021). The results were same as those from the previous pooled analysis (*X* ^2^ = 10.27 and 31.22, *p*-values = 0.0014 and <0.001 for linear and non-linear models, respectively). The relative risk of maternal hypotension was 0.85 (95% CI from 0.77 to 0.94, *p* = 0.001) with every 0.01 μg/kg/min increase in the NE infusion rate. The estimated ED_50_ and ED_95_ of NE infusion rates for hypotension prophylaxis were 0.043 (95% CI from 0.027 to 0.11) and 0.19 (95% CI from 0.12 to 0.48) μg/kg/min, respectively. No single study excessively influenced the summary estimates (Figure 5). However, there remained significant heterogeneity across these studies (*X* ^2^ = 14.54 and 29.25, *p*-values = 0.0057 and 0.0223 for linear and non-linear models, respectively). Contour-enhanced funnel plots after imputation by trimfill analysis further indicated that the non-significant studies could cause publication bias (Egger’s test, *p* = 0.001) (Figure 6), which would decrease the relative risk of maternal hypotension to 0.92 (95% CI 0.85–0.99) with every 0.01 μg/kg/min increase in the NE infusion rate. TSA showed that the cumulative z-curve intersected the upper boundary of trial sequential monitoring and achieved the RIS of n = 487 for comparing NE with the saline control (Figure 7).

#### 3.3.2 Secondary outcomes

##### 3.3.2.1 Maternal outcomes

**FIGURE 8:**
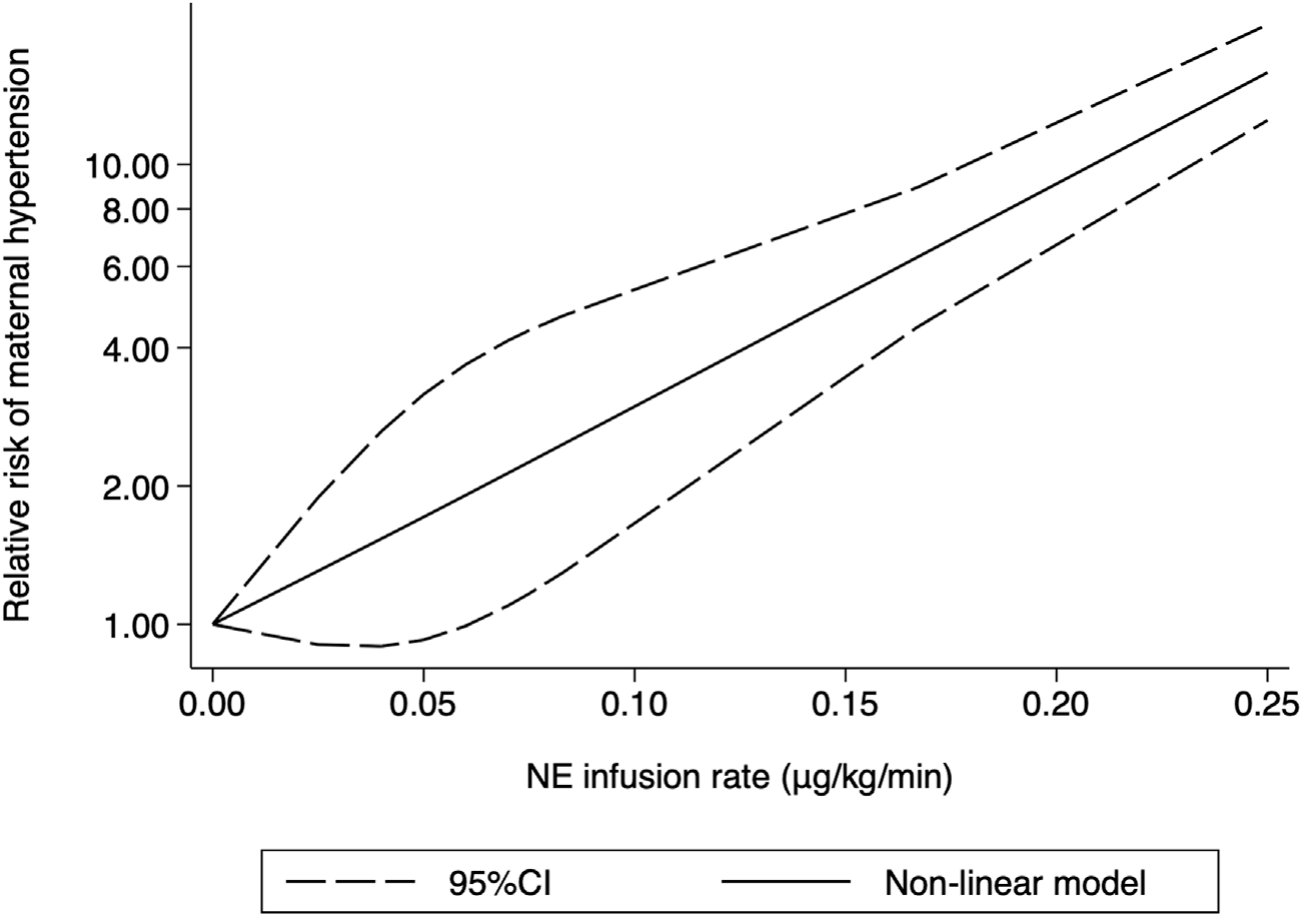
Dose–response relationship between the NE infusion rate and relative risk of maternal hypertension, showing point estimates and 95% CI for non-linear meta-analysis.

**FIGURE 9:**
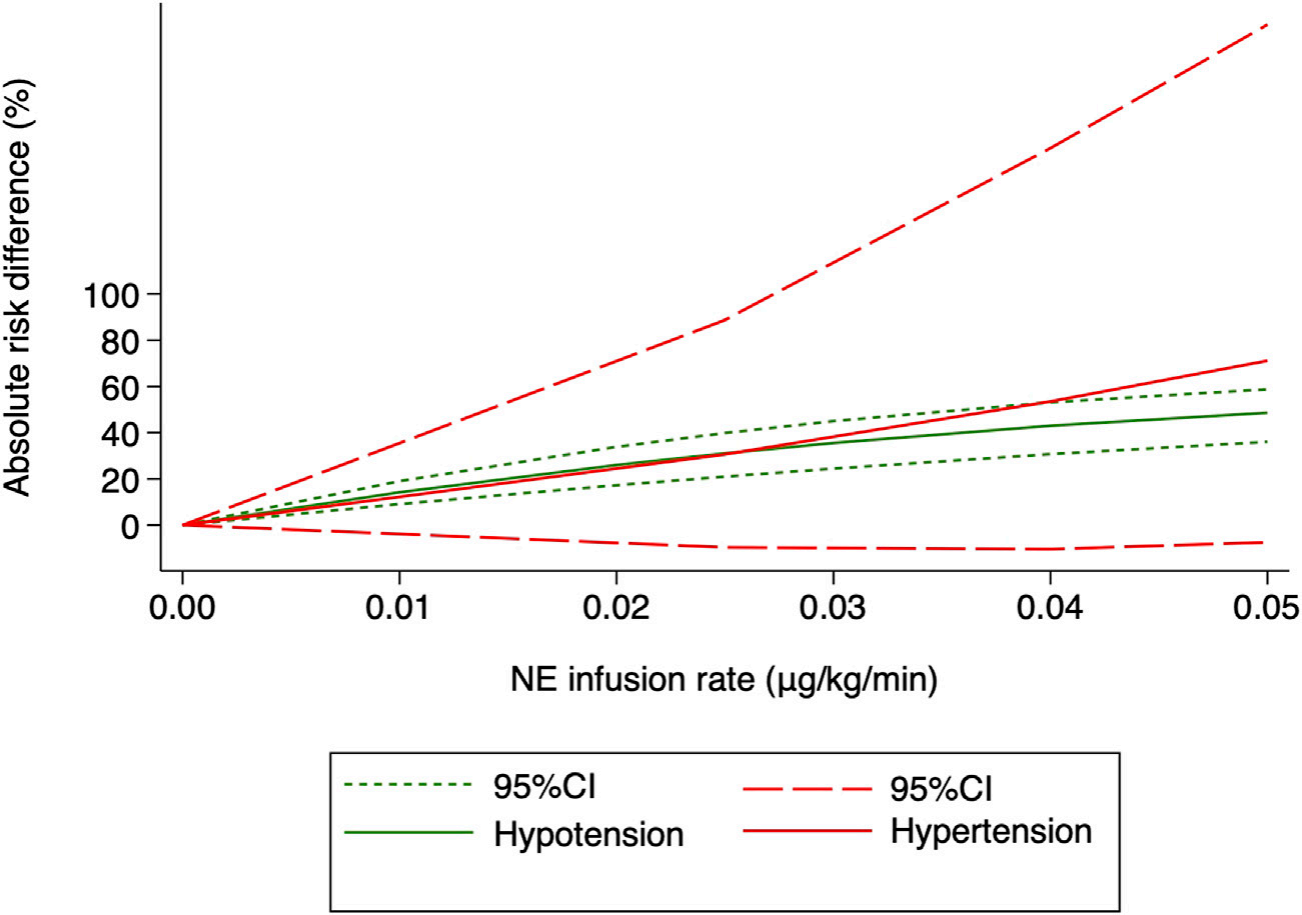
Benefit (hypotension) compared with harm (hypertension) for the NE infusion rate between 0 and 0.05 μg/kg/min. The threshold at which the benefit equaled the damage was 0.025 μg/kg/min.

**FIGURE 10:**
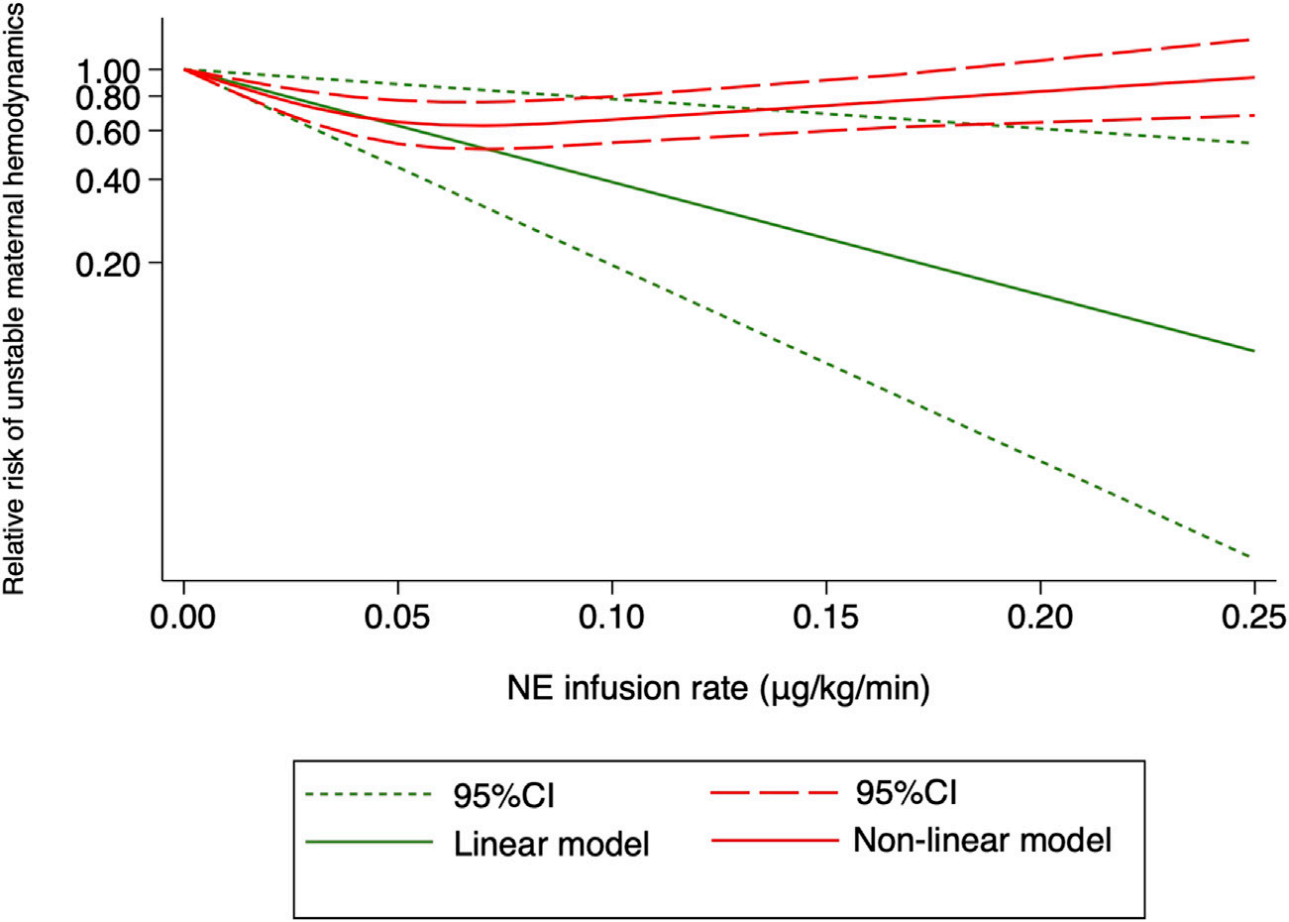
Dose–response relationship between the NE infusion rate and relative risk of unstable maternal hemodynamics, showing point estimates and 95% CI for linear and non-linear meta-analysis models.

Hypertension was defined as systolic arterial blood pressure >120% of baseline in all the 10 RCTs. Three studies (Choudhary et al., 2020; Wakhloo et al., 2021; Lyu et al., 2022) were excluded because only two different NE doses were used, and another trial (Sheng et al., 2021) was also excluded because data needed for dose–response meta-analysis were not reported. The risk of maternal hypertension increased with increased NE infusion rates (F = 256.38, *p* < 0.001) although the linear regression coefficients were insignificant (*R* ^2^ = 0.5479, *p* = 0.236) (Figure 8). The absolute risk differences for maternal hypotension and hypertension were plotted against NE infusion rates. The threshold at which the potential for benefit equaled the risk of harm was 0.025 μg/kg/min (Figure 9), with an absolute risk reduction in maternal hypotension of 31% (95% CI from 21% to 40%) and absolute risk increase in maternal hypertension of 31% (95% CI from −10% to 89%). At a larger infusion rate, the potential risk of maternal hypertension would outweigh the potential risk of hypotension. Finally, there was a U-shaped relationship between the NE infusion rate and the requirement of physician intervention for unstable hemodynamics. Parturients receiving the NE infusion rate of 0.07 μg/kg/min were at the lowest risk for physician intervention of 0.63 (95% CI from 0.51 to 0.76, Figure 10).

**FIGURE 11:**
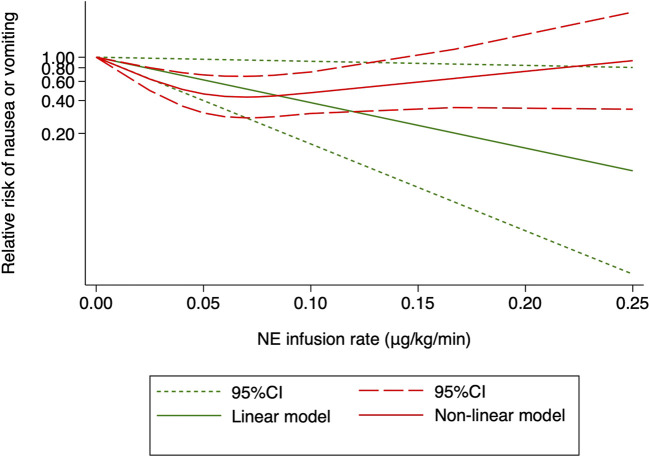
Dose–response relationship between the NE infusion rate and relative risk of maternal nausea or vomiting, showing point estimates and 95% CI for linear and non-linear meta-analysis models.

**FIGURE 12:**
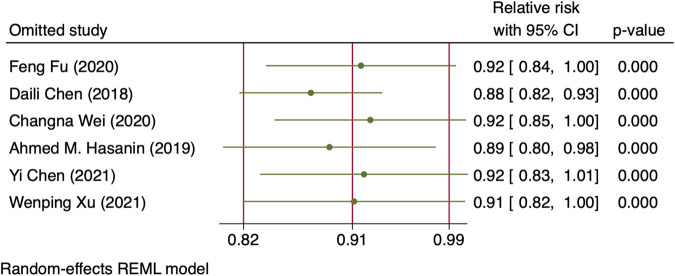
Sensitivity analysis of individual studies influenced the pooled relative risk of maternal nausea or vomiting per 0.01 μg/kg/min increase in the NE infusion rate.

**FIGURE 13:**
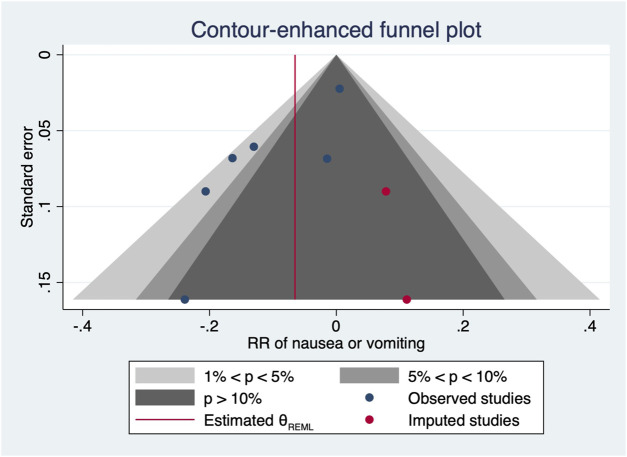
After imputation, the contour-enhanced funnel plot of maternal nausea or vomiting.

One RCT (27) was excluded for the highest versus lowest meta-analysis study for maternal bradycardia and nausea/vomiting, as it did not report the required results. A change in the NE infusion rate was not associated with any significant change in maternal bradycardia, as the relative risk was 1.013 (95% CI from 0.516 to 1.988) between the highest versus lowest NE doses. There was no heterogeneity across included trials for this outcome (I^2^ = 0%, *p* = 0.707). Meanwhile, an increased NE infusion rate was associated with the reduced risk of maternal nausea/vomiting. The relative risk of maternal nausea/vomiting between the highest versus the lowest dose of NE was 0.522 (95% CI from 0.336 to 0.811). There were mild heterogeneities among included trials for these outcomes (I^2^ = 10.6% with *p* = 0.347). In the subsequent dose–response meta-analysis, three studies (Choudhary et al., 2020; Wakhloo et al., 2021; Lyu et al., 2022) were further excluded because only two different NE doses were used, and another trial was also excluded (Sheng et al., 2021) as it did not report the required results for dose–response meta-analysis. There were positive linear and non-linear dose–response relationships between the NE infusion rate and relative risk of maternal nausea/vomiting from random effects (*X* ^2^ = 4.64 and 14.36, *p*-values = 0.0312 and 0.0008, respectively). Across these included studies, there was significant heterogeneity for the linear model (*X* ^2^ = 14.57, *p* = 0.0124) while non-significant heterogeneity for the non-linear model (*X* ^2^ = 10.98, *p* = 0.8954). The pooled relative risk of maternal nausea/vomiting was 0.91 (95% CI from 0.82 to 0.99, *p* = 0.031) with every 0.01 μg/kg/min increase in the NE infusion rate. However, with the NE infusion rate over 0.075 μg/kg/min, the risk of maternal nausea/vomiting would increase (Figure 11). No individual study excessively influenced the summary estimate (Figure 12). Contour-enhanced funnel plots after imputation by trimfill analysis found the non-significant studies that caused publication bias (Egger’s test, *p* = 0.025) (Figure 13), which would decrease the pooled relative risk of maternal nausea or vomiting to 0.937 (95% CI from 0.869 to 1.011) with every 0.01 μg/kg/min increase in the NE infusion rate.

##### 3.3.2.2 Fetal outcomes

For the highest versus the lowest dose of NE analysis, the mean difference of the change in the umbilical arterial pH value and relative risk of Apgar score <7 at 1 min was 0.243 (95% CI from −0.08–0.566) and 1.015 (95% CI from 0.353 to 2.921), respectively. These results suggested that change in NE doses or infusion rates was not associated with a significant change in fetal outcomes. In addition, there is significant heterogeneity across the trials for the arterial pH value (I^2^ = 56.9%, *p* = 0.041) while non-significant heterogeneity for Apgar score <7 at 1 min (I^2^ = 0%, *p* = 0.996).

### 3.4 Quality of evidence

The quality of evidence for the primary outcome assessed with GRADE guidelines is reported in Table 2.

**TABLE 2:** Summary of findings (SOF) for primary outcomes. Question: Per 0.01 μg/kg/min increase in the norepinephrine infusion rate compared to placebo for spinal anesthesia-induced maternal hypotension for an elective cesarean section. Setting: An operating room.

Certainty assessment							No. of patients		Effect		Certainty	Importance
No. of studies	Study design	Risk of bias	Inconsistency	Indirectness	Imprecision	Other considerations	Per 0.01 μg/kg/min increase in the norepinephrine infusion rate	Placebo	Relative (95% CI)	Absolute (95% CI)		
The incidence of maternal hypotension												
7	Randomized trials	Not serious	Serious	Not serious	Serious	Publication bias strongly suspected dose–response gradient	159/655 (24.3%)	124/224 (55.4%)	**RR 0.86** (0.80–0.92)	**78 fewer per 1,000** (from 111 fewer to 44 fewer)	⊕⊕○○ Low	Critical

**CI**, confidence interval; **RR**, risk ratio.

## 4 Discussion

In this systematic review and meta-analysis, the dose–response relationship between the NE infusion rate and the risk of maternal hypotension after spinal anesthesia was observed. With every 0.01 μg/kg/min increase in the NE infusion rate, there was a 14% decrease in the rate of maternal hypotension. For post-spinal hypotension prophylaxis, the ED_50_ and ED_95_ of NE infusion rates were estimated to be 0.046 (95% CI from 0.032 to 0.085) and 0.2 (95% CI from 0.14 to 0.37) μg/kg/min, respectively. An NE infusion rate at 0.07 μg/kg/min was associated with the lowest risk of physician intervention for unstable hemodynamics.

Fluid infusion is another important intervention for post-spinal hypotension. However, it is not worth delaying spinal injection for fluid preload as administration of a fixed volume of fluid before the spinal injection is of limited effectiveness in reducing the incidence of hypotension (Author Anonymous, 2016). In contemporary anesthetic practice, fluid administration is generally initiated with spinal anesthesia, referred to as the fluid co-load (Kinsella et al., 2018), and this is also the truth found in most trials included in this meta-analysis. In only one trial (Choudhary et al., 2020), fluid was administrated *via* pre-load alone, and it failed to demonstrate a reduced risk of hypotension with a larger dose of NE. This trial was then excluded from the later dose–response analysis. Therefore, the NE infusion rate defined in this meta-analysis was based on fluid co-load during spinal anesthesia, which is the mainstream strategy for fluid administration in current obstetric anesthesia.

As expected, a dose–response relationship was identified between the prophylactic NE infusion rate and incidence of post-spinal hypotension with our systematic review and meta-analysis. The dose–response relationship was robust, as there was no change in slope estimates after considering sensitivity analysis or publication bias. The results of TSA and included patient numbers exceeding the required information size also supported the reliability of our meta-analysis. However, significant heterogeneity was found among the RCTs, and subgroup analysis cannot eliminate heterogeneity. Therefore, confounding factors other than bolus infusion combination, such as intravenous fluid loading volume, level of sensory blockade, and doses of spinal anesthetics, should be considered.

The systematic review and meta-analysis further showed that the risk of hypertension would overweigh the risk of hypotension if the NE infusion rate was greater than 0.025 μg/kg/min. To maintain a stable hemodynamic status after spinal anesthesia, but not only to prevent hypotension, is the goal of peri-cesarean management. From this prospective, an initial NE infusion rate of 0.07 μg/kg/min is recommended as it was associated with least physician intervention for unstable hemodynamics (for both hypotension and hypertension). Our results further suggested that NE infusion at a fixed weight-adjusted infusion rate alone might not be optimal for a stable hemodynamic status following spinal anesthesia during cesarean section. Other maneuvers, such as a closed-loop infusion system or individual vasopressor responsiveness prediction system, should be integrated into our practice.

Nausea or vomiting is another common complication following spinal anesthesia for cesarean section, especially in patients experiencing hypotension (Cooperman, 1972; Balki and Carvalho, 2005). Unlike previous studies (Fu et al., 2020), the incidence of nausea or vomiting is inversely correlated with the NE infusion rate. However, the NE infusion rate over 0.075 μg/kg/min was associated with an increased, but not a decreased, incidence of nausea and vomiting. One possible explanation is that 87 (59%) patients with NE infusion rates over 0.075 μg/kg/min were extracted from one single study in which a larger NE infusion rate failed to reduce the rate of post-spinal hypotension (Chen et al., 2018). Furthermore, the small sample size and publication bias contributed to the paradoxical dose–response relationship between NE infusion rate and maternal nausea or vomiting.

Our meta-analysis found no dose–response relationship between NE infusion rates and risks of maternal bradycardia. As a pure α-adrenergic receptor agonist, phenylephrine is associated with dose-dependent reflex bradycardia, which may lead to a decreased cardiac output (CO) (Stewart et al., 2010). NE has an additional β-adrenergic receptor agonist activity, which counteracts the decrease in the heart rate following α-adrenergic receptor activation. Thus, NE increases blood pressure with little change in the heart rate, as found in this meta-analysis. Our results also showed that an increased NE infusion rate had no effect on the umbilical artery pH and Apgar scores of newborns, which could be safely used in obstetric anesthesia (Ngan Kee et al., 2020; Singh et al., 2020).

### 4.1 Limitations

Our study had several limitations. First, an NE infusion rate greater than 0.1 μg/kg/min was used in only one study (Chen et al., 2018). Therefore, the extrapolated ED_95_ of 0.2 μg/kg/min should be interpreted cautiously. Second, there was some heterogeneity among RCTs, which cannot be eliminated by subgroup analysis of different modes of NE administration. Third, our meta-analysis was only conducted with weight-adjusted, fixed-rate infusions of NE without comparing non-weight-adjusted, variable-rate infusions of NE due to a lack of access to and examination of data from individual participants. Fourth, obese patients, defined as BMI ≥40 kg/m^2^, were excluded in seven (Hasanin et al., 2019; Fu et al., 2020; Wei et al., 2020; Chen et al., 2021; Sheng et al., 2021; Wakhloo et al., 2021; Xu et al., 2021) of the 10 included trials. Therefore, the dosage identified in this dose–response meta-analysis should not be extrapolated into overweight patients directly. It remains unclear whether ideal body weight should be considered when calculating the NE infusion rate for severely obese patients, which warrants further investigation.

## 5 Conclusion

Our dose–response meta-analysis shows that every 0.01 μg/kg/min increase in the NE infusion rate is associated with a 14% decrease in the spinal anesthesia-induced maternal hypotension rate. NE’s ED_50_ and ED_95_ prophylactic infusion rates are 0.046 μg/kg/min (95% CI 0.032–0.085) and 0.2 (95% CI 0.14–0.37), respectively. Using larger doses of NE does not eliminate hypotension but causes reactive hypertension. An NE infusion rate at 0.07 μg/kg/min was recommended as the initial NE infusion rate for post-spinal hypotension prophylaxis, which was associated with the lowest risk of physician intervention for unstable hemodynamics.

## Data Availability

The original contributions presented in the study are included in the article/Supplementary Material, further inquiries can be directed to the corresponding author.
