# Supplementary material for: Prophylactic intravenous norepinephrine for the prevention of hypotension during spinal anesthesia for elective cesarean section: a systematic review and dose–response meta-analysis of randomized controlled trials
Source: Front Pharmacol. 2023 Sep 19;14:1247214. doi: 10.3389/fphar.2023.1247214 (PMC10546307; doi:10.3389/fphar.2023.1247214)
Supplement: Supplementary file 1 [file Table1.DOCX]

Supplementary Material

- Prophylactic intravenous norepinephrine for the prevention of hypotension during spinal anesthesia for elective cesarean section: a systematic review and dose-response meta-analysis of randomized controlled trials

Yuan Li, Bingxing Shuai, Han Huang^*^

*** Correspondence: Han Huang**: han.huang@scu.edu.cn

# Supplementary Data

**Appendix**

EBM Reviews - Cochrane Central Register of Controlled Trials <June 2022>

Embase <1974 to 2022 July 22>

Ovid MEDLINE(R) and Epub Ahead of Print, In-Process, In-Data-Review & Other Non-Indexed Citations, Daily and Versions <1946 to July 22, 2022>

1 exp Norepinephrine/ 217833

2 Noradrenaline*.tw. 81351

3 Levarterenol.tw. 252

4 Levophed*.tw. 1115

5 Norepinephrin*.tw. 131315

6 Arterenol.tw. 655

7 Levonor*.tw. 13913

8 1 or 2 or 3 or 4 or 5 or 6 or 7 310056

9 exp Cesarean Section/ 167448

10 Cesarean Sections.tw. 9122

11 Caesarean Section*.tw. 56908

12 Abdominal Deliver*.tw. 1090

13 C-Section*.tw. 6714

14 Postcesarean Section.tw. 188

15 9 or 10 or 11 or 12 or 13 or 14 191032

16 8 and 15 1096 (Cochrane:122, Embase:728, MEDLINE:246)

17 remove duplicates from 16 832 (Cochrane:119, Embase:525, MEDLINE:188)

18 clinical trial/ 1574664

19 randomized controlled trial/ 1293735

20 Random Allocation/ 218077

21 single-blind method/ 100168

22 Double-Blind Method/ 492790

23 cross-over studies/ 158133

24 Placebos/ 387166

25 Randomi?ed controlled trial$.tw. 736168

26 RCT.tw. 108691

27 Random allocation.tw. 6907

28 Randomly allocated.tw. 117463

29 Allocated randomly.tw. 7921

30 (allocated adj2 random).tw. 2677

31 Single blind$.tw. 82908

32 Double blind$.tw. 661484

33 ((treble or triple) adj blind$).tw. 4975

34 Placebo$.tw. 921539

35 Prospective Studies/ 1409894

36 18 or 19 or 20 or 21 or 22 or 23 or 24 or 25 or 26 or 27 or 28 or 29 or 30 or 31 or 32 or 33 or 34 or 35 4989928

37 Humans/ 38710799

38 36 and 37 3719806

39 16 and 38 252 (Cochrane:46, Embase:123, MEDLINE:83)

40 remove duplicates from 39 147 (Cochrane:45, Embase:64, MEDLINE:38)
